# Mapping of the extracellular RBP4 ligand binding domain on the RBPR2 receptor for Vitamin A transport

**DOI:** 10.3389/fcell.2023.1105657

**Published:** 2023-02-22

**Authors:** Rakesh Radhakrishnan, Matthias Leung, Ashish K. Solanki, Glenn P. Lobo

**Affiliations:** ^1^ Department of Ophthalmology, University of Minnesota, Minneapolis, MN, United States; ^2^ Department of Medicine, Medical University of South Carolina, Charleston, SC, United States; ^3^ Department of Ophthalmology, Medical University of South Carolina, Charleston, SC, United States

**Keywords:** vitamin A, RBP4, Rbpr2, STRA6, all-trans retinol

## Abstract

The distribution of dietary vitamin A/all-*trans* retinol/ROL throughout the body is critical for maintaining retinoid function in peripheral tissues and for retinoid delivery to the eye in the support of visual function. In the circulation, all-*trans*-retinol bound to the RBP4 protein is transported and sequestered into target tissues for long-term storage. Two membrane receptors that facilitate all-*trans* retinol uptake from RBP4 have been proposed. While it is well established that the membrane receptor, STRA6, binds to circulatory RBP4 for ROL transport into the eye, the second vitamin A receptor, RBPR2, which is expressed in non-ocular tissues, is less characterized. Based on the structural homology between these two RBP4 receptors, published literature, and from our recent work in *Rbpr2* ^*−/−*^ deficient mice, we hypothesized that RBPR2 might also have high-binding affinity for RBP4 and this mechanism facilitates ROL transport. Herein, we aimed to elucidate the membrane topology and putative RBP4 binding residues on RBPR2 to understand its physiological function for retinoid homeostasis. Using *in silico* analysis and site-directed mutagenesis, we identified a potential RBP4 binding domain on RBPR2. We employed an *in vitro* cell-based system and confirmed that mutations of these residues on RBPR2 affected its binding to exogenous RBP4 and subsequently vitamin A uptake. Using Surface Plasmon Resonance assays, we analyzed both the binding affinities and kinetic parameters of wild-type RBPR2 and individual mutants affecting the RBPR2-RBP4 binding domain with its physiological ligand RBP4. These studies not only revealed a putative RBP4 binding domain on RBPR2 but also provided new structural, biochemical, and critical information on its proposed role in RBP4 binding for ROL transport and retinoid homeostasis.

## Introduction

All-*trans* retinol (ROL) is the major form of vitamin A found within circulation. It is essential for normal embryonic development, reproduction, immunity, and is critical for ocular retinoid (von Lintig, 2012; Sun, 2012; Wassef and Quadro, 2011; D’Ambrosio et al., 2011). Within tissues, ROL is the precursor for all-*trans* retinoic acid (RA), an essential ligand for nuclear receptors such as the retinoic acid receptor (RAR), which regulate gene transcription. ROL is also the precursor for 11-*cis* retinaldehyde (RAL), the essential visual chromophore that isomerizes through exposure to light, and by extension allows photoreceptor cells to detect light (von Lintig, 2012; Borel and Desmarchelier, 2017). Humans cannot synthesize vitamin A *de novo*, so all vitamin A and its derivatives in the human body, collectively called retinoids, originate from dietary consumption of vitamin A precursors (von Lintig, 2012; Borel and Desmarchelier, 2017). The bulk of dietary retinoids (80%–85%) are stored within the liver as retinyl esters (RE). Under fasting conditions, the liver is responsible for maintaining retinoic homeostasis through the conversion of its RE storage into ROL, and secreting ROL bound to retinol binding protein 4 (RBP4) into the bloodstream. (Biesalski et al., 1999; Quadro et al., 1999; Kawaguchi et al., 2007; Harrison, 2012; Lobo et al., 2012; Amengual et al., 2014; Kelly and von Lintig, 2015; Borel and Desmarchelier, 2017).

For retinol to perform its biological function it must first be absorbed within cells and this requires membrane receptors specific to the complex formed by RBP4 and retinol (RBP4-ROL) (Harrison, 2012; Lobo et al., 2012). Currently, two membrane receptors that facilitate the uptake of retinol from circulatory RBP4-ROL have been proposed (von Lintig, 2012; Kawaguchi et al., 2007; Kelly and von Lintig, 2015; Amengual et al., 2014). In 2007, the Sun lab discovered the stimulated by retinoic acid 6 (STRA6) cell membrane receptor, which is proposed to transport retinol intracellularly from circulatory RBP4-ROL into target tissues, such as the eye (Kawaguchi et al., 2007). STRA6 is highly expressed in blood-organ barrier structures and organs that require high amounts of retinoid for proper function, such as the retinal pigment epithelium (RPE), reproductive organs, brain, and kidney (Amengual et al., 2014; Kelly and von Lintig, 2015) Correspondingly, Matthew-Wood Syndrome is characterized by visual abnormalities and developmental problems linked to mutations in the human *STRA6* gene (Golzio et al., 2007; Pasutto et al., 2007; Isken et al., 2008). Studies from the von Lintig lab and others have genetically confirmed in zebrafish and mouse models the importance of STRA6 for vitamin A homeostasis of peripheral tissues, where severe ocular defects were reported in animals lacking STRA6 (Isken et al., 2008; Berry et al., 2013; Amengual et al., 2014; Kelly and von Lintig, 2015). To understand the importance of STRA6 in RBP4 binding for ROL transport, the Sun lab used a large-scale mutagenesis approach and identified an essential RBP4 binding domain in STRA6, where they showed that mutations within individual amino acid residues within this RBP4 binding domain affects binding of STRA6 to exogenous RBP4, and this consequently affected ROL transport (Kawaguchi et al., 2008a; Kawaguchi et al., 2008b; Kawaguchi and Sun, 2010; Sun and Kawaguchi, 2011). From these studies, it is apparent that membrane receptors that interact with RBP4-ROL, such as STRA6, must contain one or more binding residues/domains, which are essential for receptor binding to circulatory RBP4 for ROL internalization into target tissues, such as the eye (Golzio et al., 2007; Pasutto et al., 2007; Isken et al., 2008; Kawaguchi et al., 2008a; Kawaguchi et al., 2008b; Kawaguchi and Sun, 2010; Sun and Kawaguchi, 2011; Berry et al., 2013).

Another less studied receptor for RBP4 binding and ROL transport is the retinol binding protein 4 receptor 2 (RBPR2) protein, also annotated as STRA6like (Alapatt et al., 2013; Shi et al., 2017; Lobo et al., 2018; Radhakrishnan et al., 2022a). RBPR2 was first identified by the Graham group in 2013, where they proposed its function in the regulation of retinol homeostasis in the liver and in non-ocular tissues (Alapatt et al., 2013). RBPR2 is expressed both in zebrafish and mouse liver, intestine, and other non-ocular tissues, but not in the eye. As such, RBPR2 could act as the RBP4-ROL receptor in these STRA6 lacking tissues, contributing to the maintenance of proper ocular retinoid concentrations for retinal homeostasis and visual function through the regulation of serum retinoid homeostasis (Shi et al., 2017; Lobo et al., 2018; Martin et al., 2021; Radhakrishnan et al., 2022a). Our recent study in *Rbpr2* knockout (*Rbpr2* ^*−/−*^) mice showed that under vitamin A deficient diets, *Rbpr2* ^*−/−*^ mice failed to maintain retinal function and showed decreased systemic and ocular retinoid concentrations, which manifested as photoreceptor phenotypes (Radhakrishnan et al., 2022a). These *Rbpr2* ^*−/−*^ mice also displayed an imbalance in opsin pigment synthesis and stoichiometry, resulting in decreased visual function, when compared to control mice on similar vitamin A diets (Radhakrishnan et al., 2022a). While the observed ocular phenotypic changes in mice lacking a systemic membrane receptor for RBP4-ROL are apparent, the coordination between retinol consumption in the eye for vision and retinol supply from long-term storage at the systemic level, is one of the less characterized areas in understanding retinoid homeostasis. Even less characterized is the non-ocular RBP4 receptor RBPR2 itself, its mechanisms for RBP4 binding and ROL transport in target tissues, and its role for retinoid homeostasis (Kelly and von Lintig, 2015; Borel and Desmarchelier, 2017; Solanki et al., 2020; Martin et al., 2021; Radhakrishnan et al., 2022a). In this study, we aimed to identify putative RBP4 binding residues on the membrane receptor, RBPR2, to establish the importance of these RBP4 binding residues on RBPR2 for ROL transport.

## Materials and methods

### Materials

All chemicals, unless stated otherwise were purchased from Sigma-Aldrich (St. Louis, MO, United States).

### Homology modeling and molecular docking

Online server SWISS-MODEL (http://swissmodel.expasy.org/) was used to generate homology based models of mouse RBPR2, mouse STRA6, and zebrafish Stra6. The model with maximum coverage and lowest Z score for each was selected for further studies. The template selected (by online server SWISS-MODEL) was the cryoEM structure of STRA6, receptor for retinol (PDB ID 5sy1, Chain B) which showed nearly 44% sequence identity and Q mean close to −5 for STRA6 identity; and nearly 22% sequence identity and Q mean close to −7 for RBPR2. The structure for RBP4 was obtained from PDB database (RSCB PDB ID: 2wqa, Chain E). The models generated were used for docking studies to analyze the protein-protein interactions employing the online data-driven docking program HADDOCK. HADDOCK requires a set of ambiguous interaction restraints (AIRs) at the binding interface that are divided into “active” and “passive” categories where active residues are those directly implicated in binding from experimental data and passive residues are their near neighbors. The docking process included a rigid body energy minimization step. The residues S294, Y295 and L296 for mouse RBPR2 were assigned as active residues for interaction as per the previous published papers to be essential for binding (Alapatt et al., 2013; Chen et al., 2016). The residues between 8 and 12 Å from these three residues were defined as passive. HADDOCK clustered 187 structures in 11 cluster (s), which represents 93.5% of the water-refined models HADDOCK generated for RBP4-Stra6. HADDOCK clustered 138 structures in 12 cluster(s), which represents 69.0% of the water-refined models HADDOCK generated for RBP4-RBPR22. The top cluster with the minimal haddock scores of −95.1 +/− 2.0; and lowest Z-score of −1.5 for RBP4-RBPR2 was selected for analysis (van Zundert et al., 2016; Waterhouse et al., 2018).

### Cloning of the mouse *Rbpr2* cDNA

Total RNA (∼2 μg) from liver of a 2-month-old wild-type C57/B6 mouse was reverse transcribed using the SuperScript One-Step RT-PCR for LongTemplates system (Invitrogen, Grand Island, NY). The full-length mouse *Rbpr2* cDNA was amplified by using mouse gene specific *Rbpr2* primers with the Expand High Fidelity PCR system (Roche, Indianapolis, IN, USA). The amplified *Rbpr2* cDNA product was cloned in frame into the pCDNA3.1 V5/His TOPO vector (Invitrogen, Carlsbad, CA). Appropriate construction of the wild-type *Rbpr2* plasmid in the pCDNA 3.1 V5/His TOPO vector (pRbpr2-V5) was verified by sequence analysis of both strands (GENEWIZ, USA) and by comparing the sequences to the reference mouse *Rbpr*2/Stra6like cDNA sequences deposited in Ensembl (www.ensembl.org). The WT-Rbpr2 plasmid was used as a template and mutagenic Rbpr2 primer pairs were used to engineer each of the RBP4 binding residue mutants by *in vitro* site-directed mutagenesis (Quick Change II XL: Stratagene/Agilent, Santa Clara, CA), as previously achieved (Shi et al., 2017; Solanki et al., 2020). Appropriate construction of the WT-Rbpr2 and mutant-Rbpr2 plasmids were verified by DNA sequence analysis of both strands using pCDNA3.1 vector primers (GENEWIZ, USA).

### Generation of stable cell lines expressing Rbpr2 and Rbpr2/lrat

Mouse NIH3T3 cells obtained from American Type Tissue Culture (ATCC-1658) were maintained in high-glucose DMEM supplemented with 10% FBS and 1% penicillin-streptomycin sulfate and cultured at 37°C with 5% CO_2_. NIH3T3 cells were used in this experiment as they are a well-established cell line to study the *in vitro* function of vitamin A membrane receptors for RBP4 binding and ROL transport (Amengual et al., 2014; Shi et al., 2017; Lobo et al., 2018; Solanki et al., 2020). To generate constitutively expressing mouse RBPR2 in NIH3T3 cells, parental NIH3T3 or NIH3T3/LRAT expressing cells were transiently transfected with the pRbpr2-V5 plasmid, as described previously (Solanki et al., 2020). Approximately 40 h post transfection, media was replaced to contain 400 μg/mL Geneticin (G418) selection agent. After 2 weeks of selection with G418, surviving individual cells (n = 12) were selected by placing cloning rings around each surviving cell. Each clonal cell was then carefully detached by adding 10 μL of trypsin into each clonal ring. Detached cells were transferred to 6-well culture plates containing 200 μg/mL G418 selection media. Once individual clones reached ∼80% confluence they were expanded into 100 mm dishes containing 200 μg/mL of G418 selection media. To confirm stable integration of the Rbpr2 gene and expression in these cells, we isolated total protein from each clone and subject them to western blot analysis. By using the V5-primary antibody, we detected the V5-tagged RBPR2 protein.

### Indirect immunofluorescence and confocal microscopy

Cell lines were grown on coverslips and fixed in a freshly prepared mixture of 4% formaldehyde in 1X PBS (137 mM NaCl, 2.7 mM KCl, 10 mM sodium phosphate dibasic, and 2 mM potassium phosphate monobasic, pH 7.4) for 30 min at room temperature and processed as previously described (Lobo et al., 2010; Lobo et al., 2013; Solanki et al., 2020; Solanki et al., 2021). Parental NIH3T3 cells were transiently transfected with the pRbpr2-V5 plasmid, as described previously (Solanki et al., 2020). Subcellular localization of the recombinant mouse Rbpr2-V5 in NIH3T3 cells was achieved by exposure to the anti-V5 primary antibody (which detects the V5-tagged RBPR2) followed by the anti-rabbit conjugated Alexa 488 secondary antibody staining (Invitrogen, Carlsbad, CA). Cells were examined under a Zeiss LSM 510 UV Meta confocal microscope with an HCX Plan × 40 numerical aperture 1.4 oil immersion objective lens (Zeiss, Jena, Germany). Images were acquired with the Zeiss confocal software, version 2.0. All experiments were carried out in triplicate. Approximately 55–75 cells from 7–9 fields were imaged/counted per experiment (Lobo et al., 2010; Lobo et al., 2013; Rohrer et al., 2021).

### Exogenous RBP4 binding and retinol uptake studies

RBP4 cDNA cloned into the pET3a bacterial expression vector was used to express RBP4 in *E.Coli* as previously described (Shi et al., 2017). Apo-RBP4 (100 μg) was loaded with retinol in 0.2 mL of PBS by the addition of 100 μm radiolabeled retinol (American Radiolabeled Chemicals; vitamin A alcohol [3H(N)] Retinol-labeled, adjusted to 1 μCi/nmol specific activity by the addition of cold retinol) and incubating for 1 h at room temperature and then overnight at 4°C in light-protected tubes, as previously described (Shi et al., 2017). Stable NIH3T3 cells expressing either, RBPR2 or RBPR2 and LRAT were plated in 10 cm dishes. Cells were grown to 70% confluence, washed thrice with 1x PBS and incubated for 1 h in serum-free medium, at which point [^3^H]ROL-RBP4 was added for 60 min. Cells were washed thrice with 1x PBS and lysed in PBS containing 1% Nonidet P-40. Lysates were homogenized and transferred to scintillation tubes for scintillation counting. Parental NIH3T3 incubated with [^3^H]ROL-RBP4 served as controls. The RBP4-ROL binding and uptake assay was repeated thrice, using stable cells from a different passage.

### Expression and purification of human RBP4

Human RBP4 expression and purification from *Escherichia coli* was accomplished essentially as described previously (Shi et al., 2017). Briefly, human RBP4 (hRBP4) cDNA was cloned into a pET3a expression vector and expressed in BL-21 DE3 cells according to a standard protocol. Bacterial cells were harvested and lysed by osmotic shock. Insoluble material was pelleted by centrifugation, washed, and solubilized in 7M guanidine hydrochloride and 10 mM dithiothreitol. After overnight incubation, insoluble material was removed by ultracentrifugation, and the supernatant was used for the hRBP4 refolding procedure. hRBP4 was refolded by the dropwise addition of solubilized material into a mixture containing 150 μCi of [11,12-^3^H]ROL ([^3^H]ROL) (PerkinElmer Life Sciences) and non-radiolabeled ROL (Sigma) at a final concentration of 1 mm. Refolded holo-hRBP4 was dialyzed against 10 mM Tris/HCl buffer, pH 8.0, and loaded onto a DE53 anion exchange chromatography column (Whatman, Piscataway, NJ). Holo-hRBP4 was eluted with linear gradient of NaCl (0–1M) in 10 mM Tris/HCl buffer, pH 8.0. Collected fractions were examined by SDS-PAGE and UV-visible spectroscopy to ensure a proper protein/retinoid ratio. Fractions containing at least 90% holo-hRBP4 were pooled together and concentrated in a Centricon centrifugal filter device (cut-off 10,000 Da) (Millipore, Billerica, MA) to 5 mg/mL. [^3^H]ROL was quantified in a scintillation counter (Beckman Coulter, Indianapolis, IN). Holo-hRBP4 aliquots were stored at −80°C until used.

### Western blotting

Total proteins from cells were extracted using the M-PER protein lysis buffer (ThermoScientific, Beverly, MA, United States) containing protease inhibitors (Roche, Indianapolis, IN, United States). Approximately 25 μg of total protein was electrophoresed on 4%–12% SDS-PAGE gels and transferred to PVDF membranes. Membranes were probed with primary antibodies against EGFR (1:1,000; ThermoFisher/Invitrogen, Waltham, MA), HSP90 (1:2500; Invitrogen, Waltham, MA), V5 (1:2500; Sigma/Millipore, Burlington, MA), Rbp4 (1:1,000; Proteintech/Fisher Scientific, Pittsburg, PA), or β-Actin (1:10,000, Sigma) in antibody buffer (0.2% Triton X-100, 2% BSA, 1X PBS). HRP-conjugated secondary antibodies (BioRad, Hercules, CA, United States) were used at 1:10,000 dilution. Protein expression was detected using a LI-COR Odyssey or ChemiDoc Bio-Rad system, and relative intensities of each band were quantified (densitometry) using ImageJ software version 1.49 and normalized to their respective loading controls. Each western blot analysis was repeated thrice.

### Co-immunoprecipitation assays to determine binding of RBP4 to RBPR2

Co-immunoprecipitation (Co-IP) assays were performed with exogenous applied human RBP4 protein in NIH3T3/LRAT cells stably expressing V5-tagged WT-RBPR2 or individual V5-tagged RBPR2-RBP4 mutants. Using a well-established Co-IP protocol that determined extracellular STRA6-RBP4 interactions, we added reduced serum medium (8 mL of OptiMEM) containing 12 μM of purified and crosslinked T7 tagged-RBP4 to the cells and incubate this reaction for 60 min (Kawaguchi et al., 2007). After binding purified T7 tagged-RBP4 protein conjugated with the cross-linker to cells expressing WT or mutant RBPR2, followed by ultraviolet (UV) cross-linking, and membrane solubilization, cells were collected, washed thrice with 1x PBS to remove any un-bound hRBP4. Total protein was isolated and subjected to co-immunoprecipitation analysis using an RBP4 antibody, followed by reciprocal western blotting for RBPR2 (using a V5 antibody).

### Mouse RBP4 expression, purification, and quality check by circular dichroism spectroscopy and intrinsic tryptophan fluorescence assay

Recombinant mouse RBP4 with 6XHis Tag was expressed *E.coli* expression system and extracted in Tris buffer with composition of 50 mM Tris-HCl, 1 M L-Arginine, 10% Glycerol, pH 8.0. The lysate was purified by nickel NTA column. The msRBP4 protein quality was monitored by western blot using anti His-tag antibody. The structural quality of the recombinant RBP4 protein was confirmed with Circular dichroism (CD) spectroscopy (Jasco 815 circular dichroism, Spectramax Gemini) (Micsonai et al., 2015). The mean residue ellipticity (θ), was calculated using the following formula.

$$\left[\theta \right]=\left(S\times mRw\right)/\left(10cl\right)$$

where S represents the CD signal in mθ, mRw represents the mean residue mass, c represents the concentration of the protein in mg/mL, and l represents the path length in cm. The percent change in molecules structure were calculated using BeStSel Secondary Structure Analysis to Protein Fold Prediction by CD Spectroscopy (https://bestsel.elte.hu), (see Supplementary Information Supplementary Materials S1–S5). The initial interaction quality of the recombinant RBP4 with msSTRA6, msRBPR2, and control peptides were checked with intrinsic tryptophan fluorescence assay. The peptides were diluted in various micromolar concentrations and the incubated with 3 μg RBP4 in room temperature for 5 min and excited at 290 nm and the emission was scanned from 300 nm to 400 nm wavelength. The data were normalized with the blank and peptide only conditions and plotted in GraphPad prism version 9.3. San Diego, CA, United States. (Supplementary Figure S5).

### RBPR2-RBP4 binding assays using surface plasmon resonance (SPR) analysis

Purified RBP4 protein with >90% purity and 0.56 mg/mL concentration was immobilized on Biacore Sensor Chip CM5 (ITDD Biacore S200 Surface Plasmon Resonance instrument at University of Minnesota). The two-flow cell surface activated for using one as blank and other as test. Using Amine Coupling Kit (Cat. No. BR100050; Cytiva, Marlborough, MA, US) 1-ethyl-3-(3-dimethylaminopropyl) carbodiimide hydrochloride (EDC), N-hydroxysuccinimide (NHS), after surface activation, the purified RBP4 with immobilization buffer 10 mM Sodium acetate, pH 5.0, was immobilized with target of 1,200 Response Unit (RU) for achieving Rmax of 30RU in kinetic study. The reaction stopped and washed with Ethanolamine. The system was re-primed with running buffer PBST (phosphate-buffered saline solution with a 0.05% Tween20 detergent solution). The kinetic assay performed on the two flow cells, the blank was used as reference cell and the active cell with RBP4 was used for the binding study. The mouse and zebrafish RBPR2, mouse RBPR2 mutants affecting the “SYL” binding domain, and mouse STRA6 peptides (all containing the predicted RBP4 “SYL” binding residues) were chemically synthesized by Biomatik Corporation, Kitchener, ON, Canada. The peptides were serial diluted in running buffer with range of 0.8–26.6 μM and following parameter was run with contact time: 120 s, flowrate 30 μL/min, Dissociation time 300 s, Regeneration with Glycine-HCl, pH 2.5, contact time 30 s flowrate 30 μL/min and temperature 25°C. The program was run and non-specific binding on the reference cell subtracted bulk refractive index from the active sensorgram and analyzed for the association, dissociation and stabilization of the reads. The plot fitted with 1:1 binding program in Biacore™ Insight Evaluation Software, and the Graph, binding affinity plot, was plotted in GraphPad prism version 9.3.

## Results

### Mouse RBPR2 contains consensus RBP4 binding residues

**FIGURE 1:**
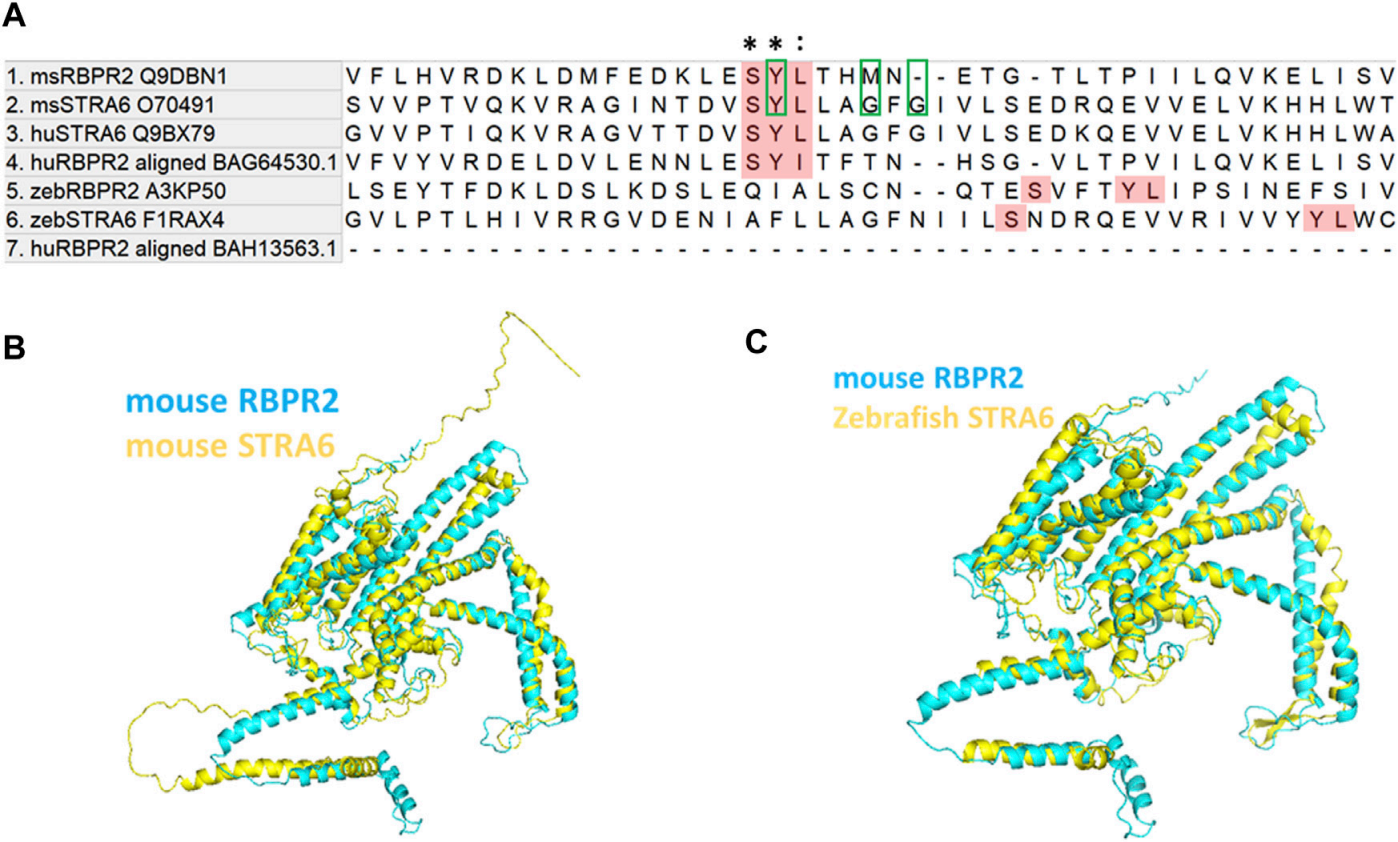
Proposed RBP4 binding residues on RBPR2 are conserved across species. **(A)** Proposed RBP4 binding residues on RBPR2 (highlighted Red; amino acid residues S294, Y295 and L296) occurs in exon 11 of mouse RBPR2 (Alapatt et al., 2013). The multiple sequence alignment of mouse and human STRA6, RBPR2 sequences shows conserved residues (Tamura et al., 2021). * indicates conserved residues of the RBP4 binding motif; (colon) indicates strongly similar properties. The previously proposed RBP4 binding residues on mouse STRA6 (Tyrosine Y336, Glycine G340, and Glycine G342) are highlighted with green box. The zebrafish STRA6 and RBPR2 sequence were not highly conserved but surprisingly had an exact topological feature alignment with the mouse sequence to extracellular region of the receptor (https://www.uniprot.org/uniprotkb/Q9DBN1/entry#sequences). **(B,C)** Computer modeling and structure homology between RBPR2 (blue) and STRA6 (yellow) proteins.

Comparison of mouse (*Ms*) RBPR2 protein sequences to human (*Hs*) STRA6 and *Ms* STRA6 revealed several short amino acid segments with >40% amino acid homology, suggesting analogous roles for these residues in the function or structural integrity of these two proteins (Figures 1A–C) (Kawaguchi et al., 2008a; Kawaguchi et al., 2008b; Kawaguchi and Sun, 2010; Sun and Kawaguchi, 2011; Alapatt et al., 2013). Interestingly, a three amino acid consensus was found in the proposed RBP4 binding domain of *Hs*. And *Ms*. STRA6, which was also found to be partially conserved in mouse and zebrafish RBPR2. The proposed RBP4 binding residues in mouse RBPR2 correspond to amino acids Serine294, Tyrosine295, and Leucine296 (SYL) (Figure 1A), which have previously been shown to be required for vitamin A transport to the eye, in zebrafish (Shi et al., 2017; Lobo et al., 2018; Solanki et al., 2020).

### Protein-ligand structural analysis confirms the importance of the proposed RBP4 binding residues on RBPR2 for ROL transport

**FIGURE 2:**
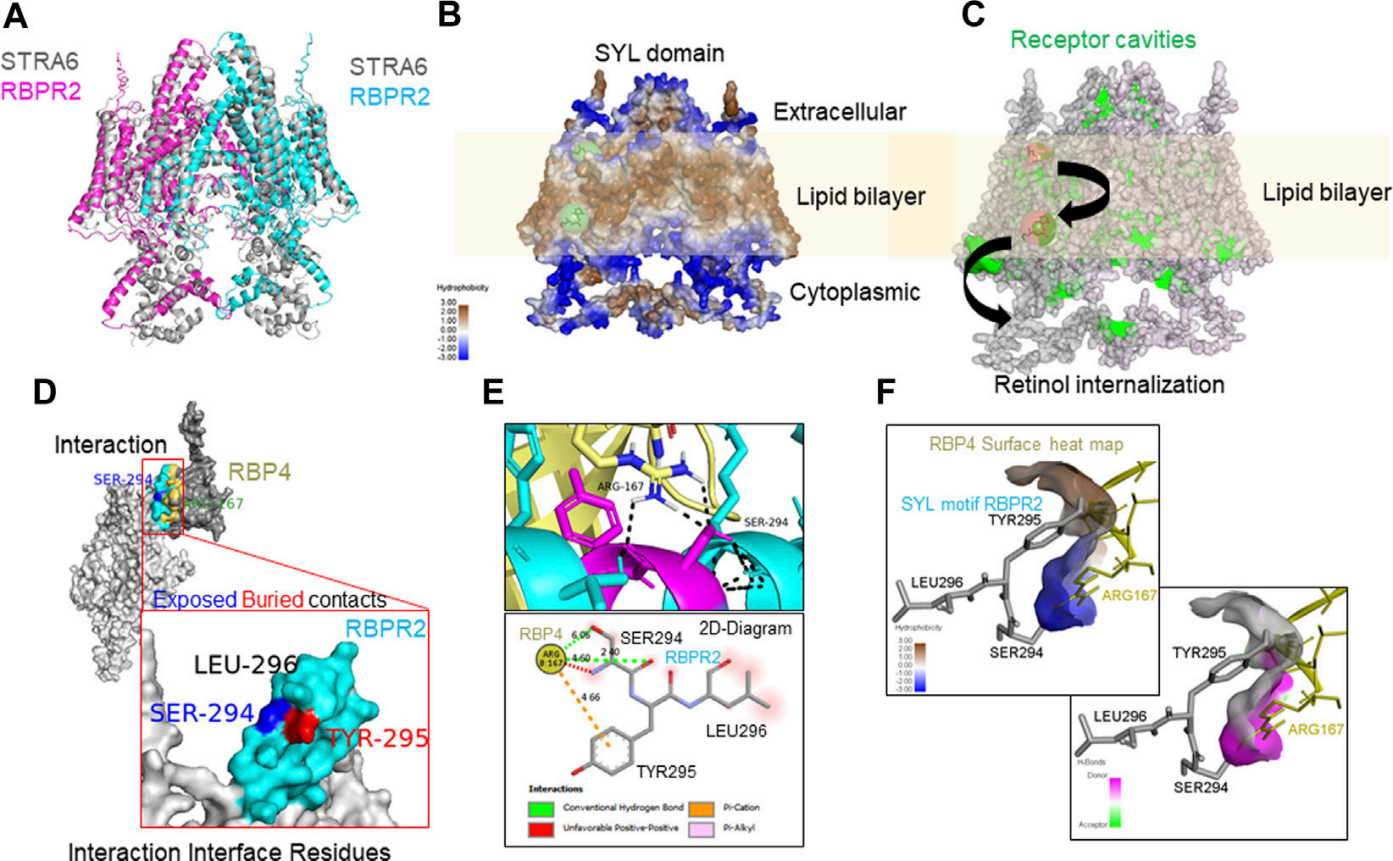
Molecular docking analysis of RBPR2-RBP4 protein interaction. **(A)** RBPR2 structural alignment with STRA6 dimer (pdb 5SY1). **(B)** Heat map indicating varying degrees of hydrophobicity within RBPR2, showing the lipid bilayer embedded regions in a RBPR2 dimer complex. The retinol binding prediction on RBPR2 dimer was performed by PyRX showing the possible regions of binding and internalization of retinol from extracellular matrix to cytosol, by utilizing the receptor cavities indicated in green on the right diagram (ref BIOVIA® Discovery Studio Visualizer v21.1). **(C)** The docking of RBPR2 monomer (Light grey) and RBP4 (Dark grey) structure showing the interactions. The interaction interface residues are color-coded, Cyan for RBPR2 residues and Yellow for RBP4. To annotate the positional exposed and buried residues information the SER-294 blue and TYR-295 Red color coded. **(D,E)** The interaction of residues and 2D-Diagram showing the mode of interactions by Hydrogen bonds, Pi-Cation, Pi-Alkyl and solvent accessible surface in Red shade. **(F)** The surface heat map of hydrophobicity and hydrogen bond of RBP4 surface showing the SYL motif of RBPR2 interacting in the pocket, analyzed by BIOVIA® Discovery Studio Visualizer v21.1.

To determine the importance of proposed RBP4 binding residues on mouse RBPR2, we first generated homology-based models of mouse RBPR2 and human STRA6 using the online server SWISS-MODEL (http://swissmodel.expasy.org/), using the cryogenic electron microscopy (cryoEM) structure of zebrafish STRA6 (PDB ID: 5sy1, Chain B) (Chen et al., 2016; van Zundert et al., 2016; Waterhouse et al., 2018), and human RBP4 (PDB ID: 2wqa, Chain E) (Figures 1B, C; Figures 2A, B). The models generated were then utilized in docking studies to analyze the STRA6-RBP4 and RBPR2-RBP4 protein-ligand interactions (docking program HADDOCK2.2) (Chen et al., 2016; van Zundert et al., 2016; Waterhouse et al., 2018). While the *in silico* binding models are assumptions, this analysis showed that the proposed and conserved residues SYL on mouse RBPR2 are part of an extracellular loop that likely plays a critical role in their interaction with RBP4 (Figures 2C–F) and thus stabilizing the complex interface with hydrophobic and hydrophilic contact. The 2D-Diagram shows the complex stabilized by the Conventional Hydrogen bond from Ser294 RBPR2, Pi-Cation and Pi-Alkyl from Tyr295 RBPR2 interacting with the Arg167 on RBP4. Leu296 on RBPR2 was not directly involved in the interaction interface, but could play an essential role in stabilizing the interaction with RBP4.

### RBPR2-mutants targeting the proposed RBP4 binding residues show normal trafficking to the plasma membrane

**FIGURE 3:**
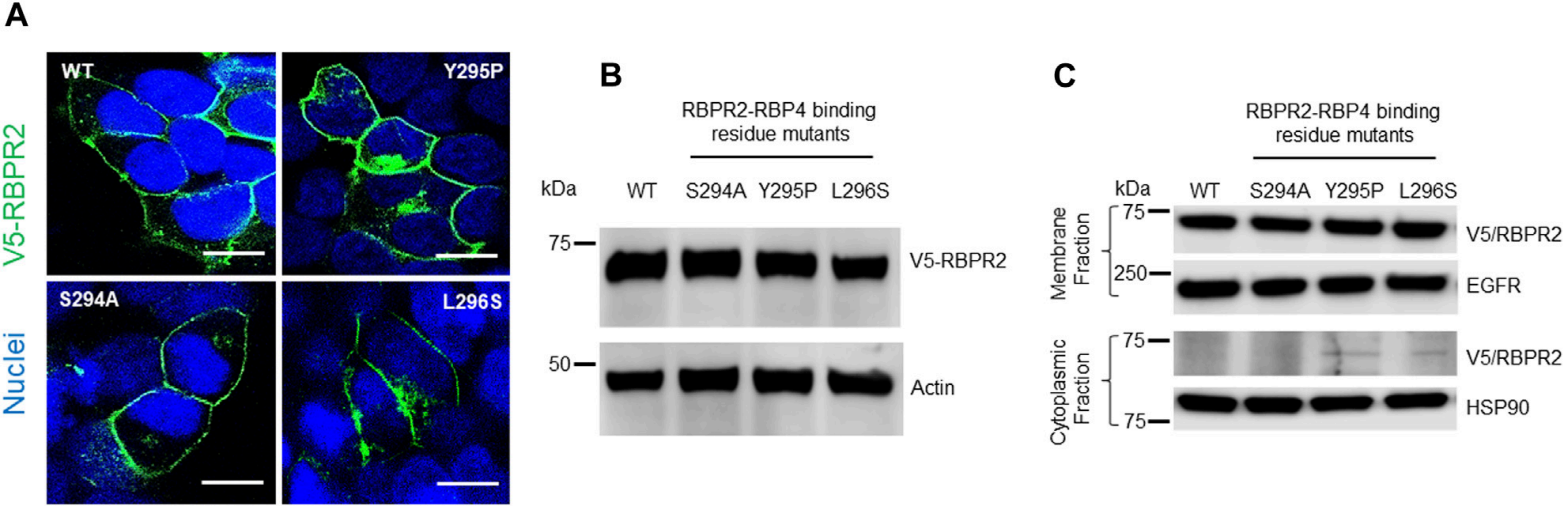
Cellular localization and expression of WT-RBPR2 and RBPR2-mutants.**(A)** Transient expression and staining of mouse V5-tagged WT-RBPR2 and variants in NIH3T3 cells using the V5-antibody. Nucleus, DAPI, blue; RBPR2-V5, Green. Scale bar = 50 μm. **(B)** Protein expression and representative western blot images of WT-RBPR2 and RBPR2-mutant proteins, which affect the “SYL” domain; anti-Actin = protein loading control. **(C)** Subcellular fractionation of stable cells expressing WT-RBPR2 or individual RBPR2-RBP4 mutants. Stable NIH3T3/LRAT cells expressing either WT-RBPR2 or individual RBPR2-RBP4 binding residues mutants were fractionated as outlined in the methods. Normalized portions of each extract (∼30 μg) were analyzed by Western blotting using antibodies against proteins from cytoplasmic (HSP90) and plasma membrane (EGFR).

To study the importance of RBP4 binding residues on RBPR2, we used site-directed mutagenesis to individually alter these putative binding residues on mouse RBPR2. Using the wild-type (WT) RBPR2-pCDNA3.1-V5 tagged vector as a template, the polar amino acids (Ser294 and Tyr295) were mutated to hydrophobic amino acids (Ser294Ala and Tyr295Pro), while the hydrophobic amino acid (Leu296) was mutated to a polar amino acid (Leu296Ser) (Kawaguchi et al., 2008a; Kawaguchi et al., 2008b). WT-RBPR2 and individual RBPR2-mutants were transiently transfected into NIH3T3 cells, and at 72 h post-transfection, WT and mutant RBPR2 expressing cells were subjected to both immunostaining and western blot analysis using the V5-antibody. Confocal microscopy analyses revealed that similar to WT-RBPR2 protein, all three single RBPR2-RBP4 binding residue mutants trafficked properly to the plasma membrane in transiently transfected NIH3T3 cells (green = V5-tagged RBPR2) (Figure 3A). Western blot and densitometry analysis further revealed that like WT-RBPR2, all three single RBPR2-mutants were equally expressed (Figure 3B). To confirm the specific subcellular localization of WT and mutant RBPR2 proteins, we subjected the transfected cells to subcellular fractionation. This analysis confirmed that individual RBPR2-mutants, like WT-RBPR2 protein, localized predominantly within the plasma membrane fractions, with only two RBPR2-mutants showing minimal cytoplasmic retention (<2% of total fractionated protein), indicating that individual RBPR2 mutants, like WT-RBPR2, trafficked properly to the plasma membrane and was expressed in this fraction (Figures 3A, C).

### RBPR2 mutants targeting the RBP4 binding sites are defective in extracellular RBP4-ROL uptake

**FIGURE 4:**
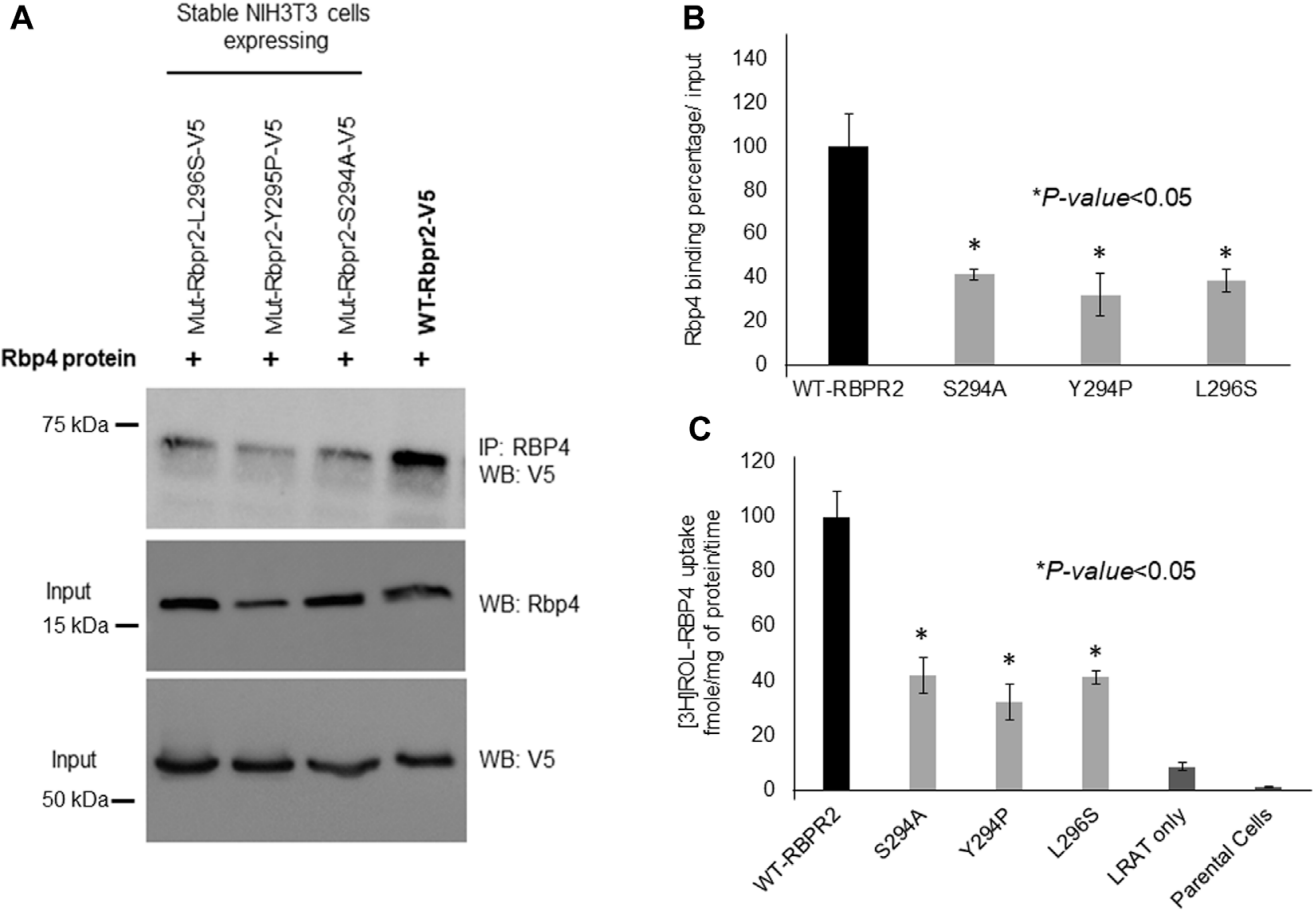
Extracellular RBP4 binding capabilities of RBPR2 and Vitamin A uptake assays. **(A,B)** Co-IP experiments showed a strong interaction between wild type (WT) RBPR2 and exogenous applied human RBP4 protein. Conversely, mutants targeting the proposed RBP4 binding sites on RBPR2, showed weaker interaction with RBP4 (62%–73% decreased binding capability compared to WT-RBPR2; *p* < 0.05). **(C)** Compared to NIH3T3/LRAT/WT-RBPR2 expressing cells, all NIH3T3/LRAT/RBPR2-mutant expressing cells showed decreased ability (<81% of WT-RBPR2 activity; *p < 0.05) to uptake extracellular applied [^3^H]ROL bound RBP4.

To determine the importance of proposed RBP4 binding residues on RBPR2 for RBP4 binding and ROL transport, we generated stable NIH3T3/LRAT cells expressing WT-RBPR2 or individual RBPR2-mutants (Shi et al., 2017; Lobo et al., 2018). Using both V5 antibody, we first confirmed equal expression of all recombinant proteins in stable cells (Figure 4A). To determine the interaction of RBPR2 with exogenous RBP4, we performed Co-IP experiments. Stable NIH3T3/LRAT cells expressing WT or individual RBPR2 mutants were seeded in 10 cm dishes. Upon reaching ∼70% confluence, a reduced serum medium (8 mL of OptiMEM) containing 12 μM of purified RBP4 was added to the cells and incubated for 120 min. Cells were collected, and total protein was isolated and subjected to co-immunoprecipitation analysis using an RBP4 antibody, followed by reciprocal western blotting for RBPR2 (using a V5 antibody). While cells expressing WT-RBPR2 showed strong binding to exogenous RBP4 protein, individual mutant RBPR2-expressing cells showed decreased binding to RBPR2 (*p* < 0.005) (Figures 4A, B). To confirm this observation, individual stable cell lines were incubated with [^3^H]ROL-RBP4 and analyzed for their ability to uptake extracellular [^3^H]ROL-RBP4 at the 60-minute time point through Liquid Scintillation Counting (Shi et al., 2017; Lobo et al., 2018). This analysis showed that control cells (NIH3T3 and NIH3T3/LRAT cells) displayed insignificant levels of [^3^H]ROL-RBP4 uptake (Figure 4C). However, [^3^H]ROL was evident in cells expressing WT-RBPR2 with co-expressed LRAT (Figure 4C). In contrast, individual RBPR2-mutant expressing cells showed significantly reduced ability (<81% decreased activity compared to WT-RBPR2; *p* < 0.005) to uptake [^3^H]ROL-RBP4, indicating that the amino acids Ser294, Tyr295, and Leu296 likely encompass the RBP4 binding residues on RBPR2 that would be crucial for ROL transport (Figure 1). Based on proper membrane trafficking of mutant RBPR2 protein (Figure 3A), but with decreased RBP4 binding (Figures 4A, B) and [^3^H]ROL-RBP4 uptake capabilities (Figure 4C), indicates the importance of these residues on RBPR2 for extracellular RBP4 interaction/binding, which is in turn critical for ROL transport.

### Surface plasmon resonance (SPR) analysis reveals binding kinetics of RBPR2 with its proposed ligand RBP4

**FIGURE 5:**
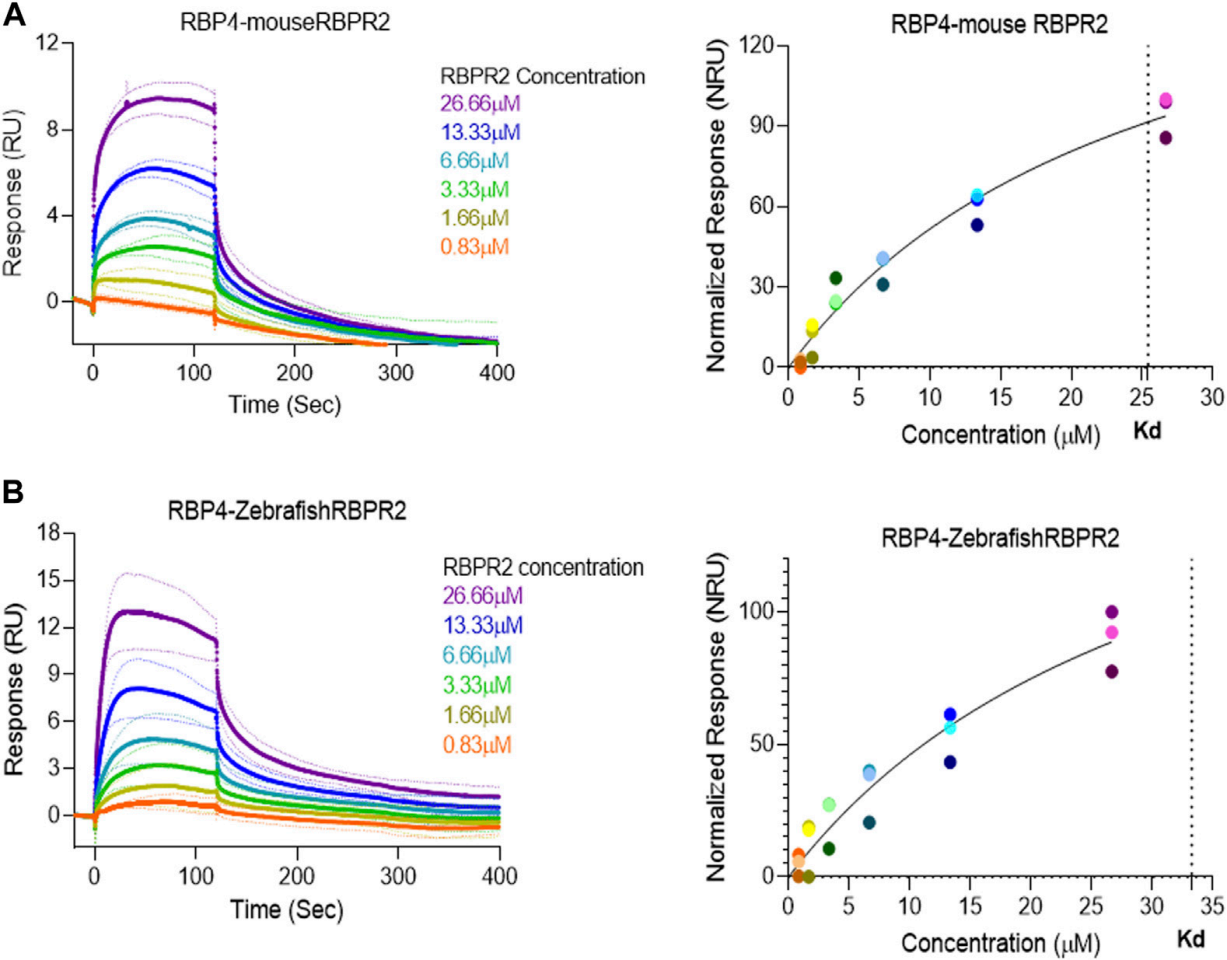
Surface Plasmon Resonance binding studies between RBPR2 and its ligand RBP4.Binding studies using SPR between mouse RBPR2 **(A)** and Zebrafish RBPR2 **(B)** and immobilized mouse RBP4 protein is shown, together with the respective kinetic values (*K*_*d*_) in the right panel. The interaction levels are measured in response units (RU) and real time plot sensorgram display the dynamics of the analysis.

**FIGURE 6:**
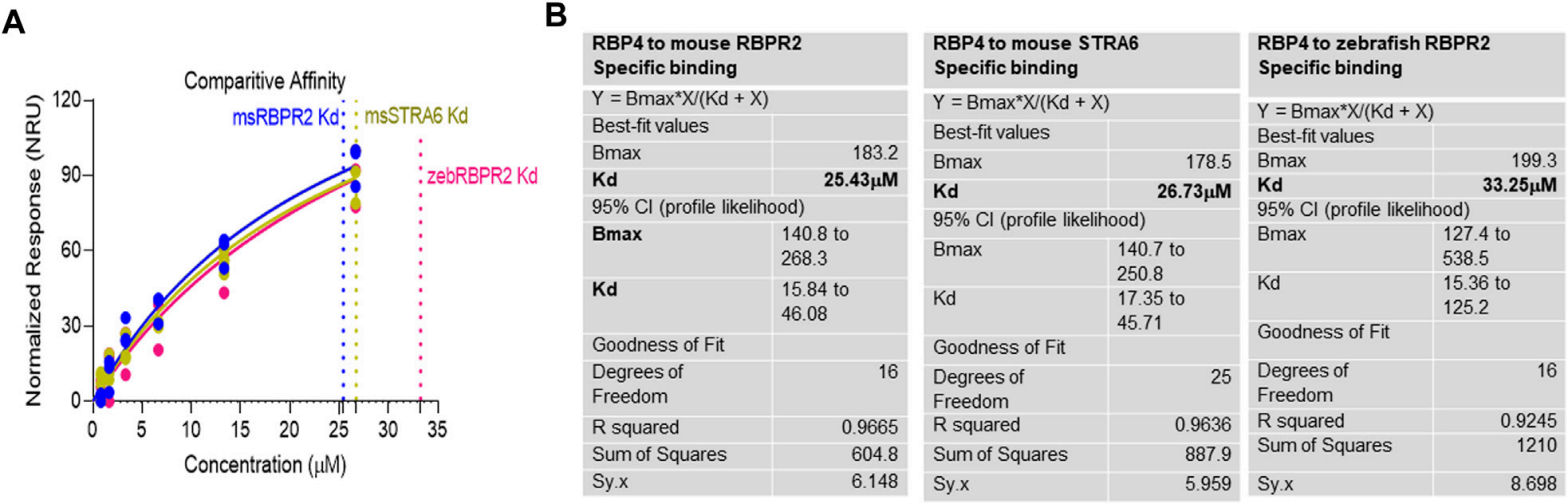
Kinetics of surface plasmon resonance binding studies between RBPR2 and its ligand RBP4. **(A,B)** Detailed SPR kinetics shown for mouse and zebrafish RBPR2 and for mouse STRA6 with its immobilized ligand mouse RBP4 protein. The affinity *K*_*d*_ values are shown in bold.

**FIGURE 7:**
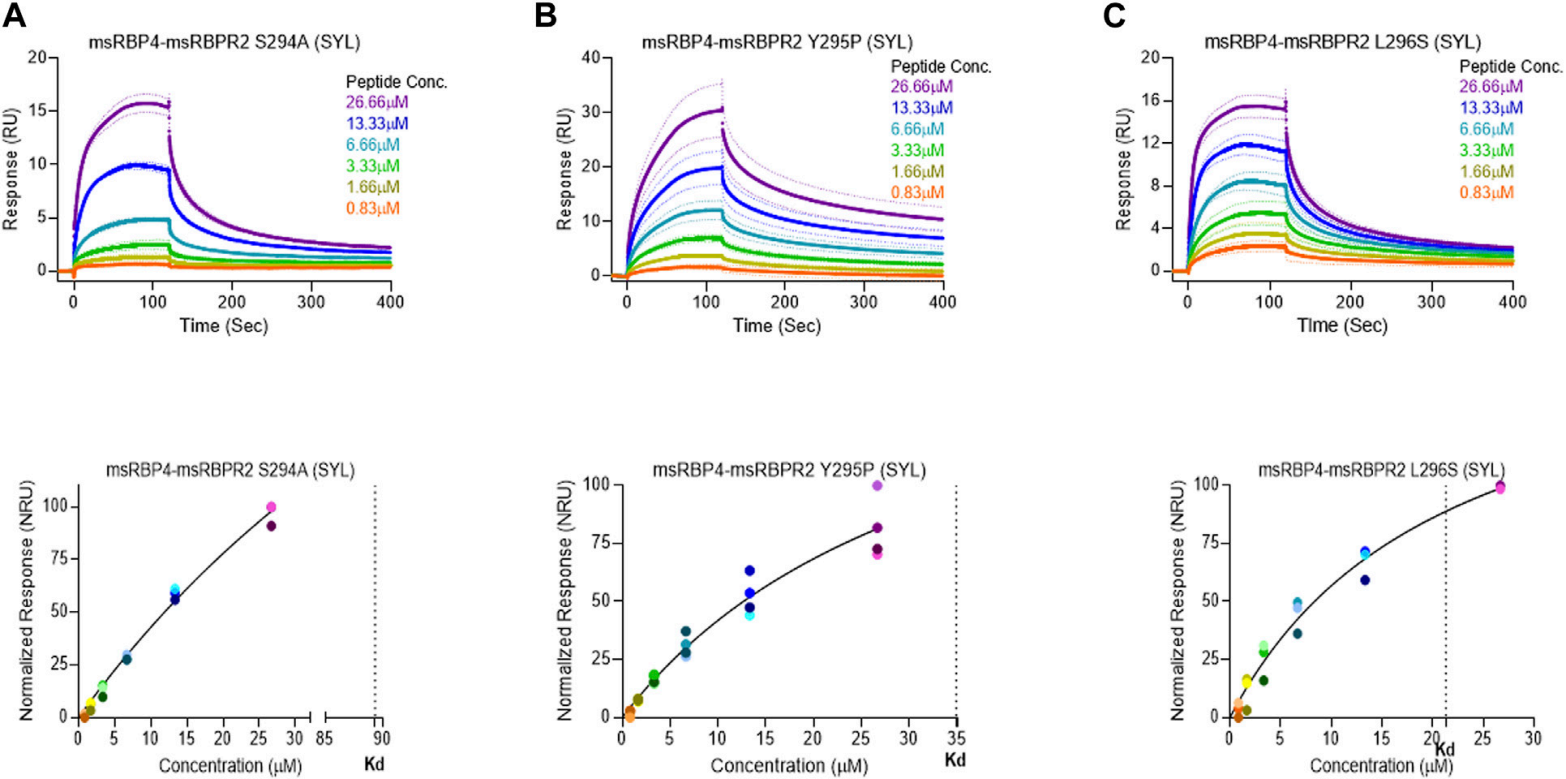
Surface plasmon resonance binding studies between mutant RBPR2 and its ligand RBP4. Binding studies using SPR between mouse RBPR2-S294A **(A)**, RBPR2-Y295P **(B)**, RBPR2-L296S **(C)** and immobilized mouse RBP4 protein is shown, together with the respective kinetic values (*K*_*d*_) in the bottom respective panels. The interaction levels measured in Response Units (RU) and real time plot sensorgram display the dynamics of the analysis.

Surface Plasmon Resonance (SPR) is a common technique used to study protein-ligand interactions. SPR can measure the binding affinities and association/dissociation kinetics of protein to ligand complexes in real-time. The interaction levels measured in Response Units (RU), and real-time plot sensorgram displays the dynamics of the analysis. The purified mouse Retinol Binding Protein, RBP4 (Supplementary Figure S1), was immobilized as the ligand, and various concentrations of the SYL motif containing peptides as analytes were run to measure SPR affinity and kinetics. The analytes examined were mouse RBPR2, zebrafish RBPR2, and mouse STRA6, known to interact with the mouse RBP4 ligand (Supplementary Figures S2–S5). To determine the *K*_*d*_ (ligand concentration in which half of the total receptor sites are occupied), the site-specific binding fitting model described below was used.

$$Y=\frac{Bmax*X}{Kd+X}$$

where B_max_ represents the maximum number of binding sites (Response Unit/RU), X represents the analyte concentration, and Y represents the binding affinity (Response Unit). Interestingly, the binding affinity of mouse RBPR2 to RBP4 approximates to the affinity of mouse STRA6 peptides to RBP4. The *K*_*d*_ of WT-*ms*RBPR2 peptide with *ms*RBP4 was 25.42 ± 6.01 μM, and B_max_ was 183.17 ± 25.66 μM (mean ± S.E). The *K*_*d*_ of *ms*STRA6 peptide with *ms*RBP4 was 26.73 ± 5.67 μM, and B_max_ was 178.45 ± 22.26 (mean ± S.E) (Figures 5, 6). The difference between the binding affinities and dissociation rate (*K*_off_) of *ms*RBPR2 and *ms*STRA6 to *ms*RBP4 was not statistically significant (*p* < 0.99) in an unpaired *t*-test, suggesting similar *K*_*d*_ values and binding affinities of these two proteins for its extracellular ligand (Supplementary Figure S6). SPR analysis was then performed on SYL mutant mouse RBPR2 with its physiological ligand RBP4. This analysis showed that RBPR2 mutants (S294A and Y295P), had higher *K*_*d*_ values of 89.33 μM and 34.91 μM respectively, while the RBPR2 mutant (L296S) had a lower *K*_*d*_ value of 21.30 μM, compared to *K*_*d*_ value of 25.42 μM for WT-RBPR2, dissociation rate (*K*_off_) for mutant msRBPR2 was significantly lower, suggesting a tighter bond formation between the RBP4 protein and mutant peptides (Figures 7A–C; Supplementary Figure S7).

## Discussion

**FIGURE 8:**
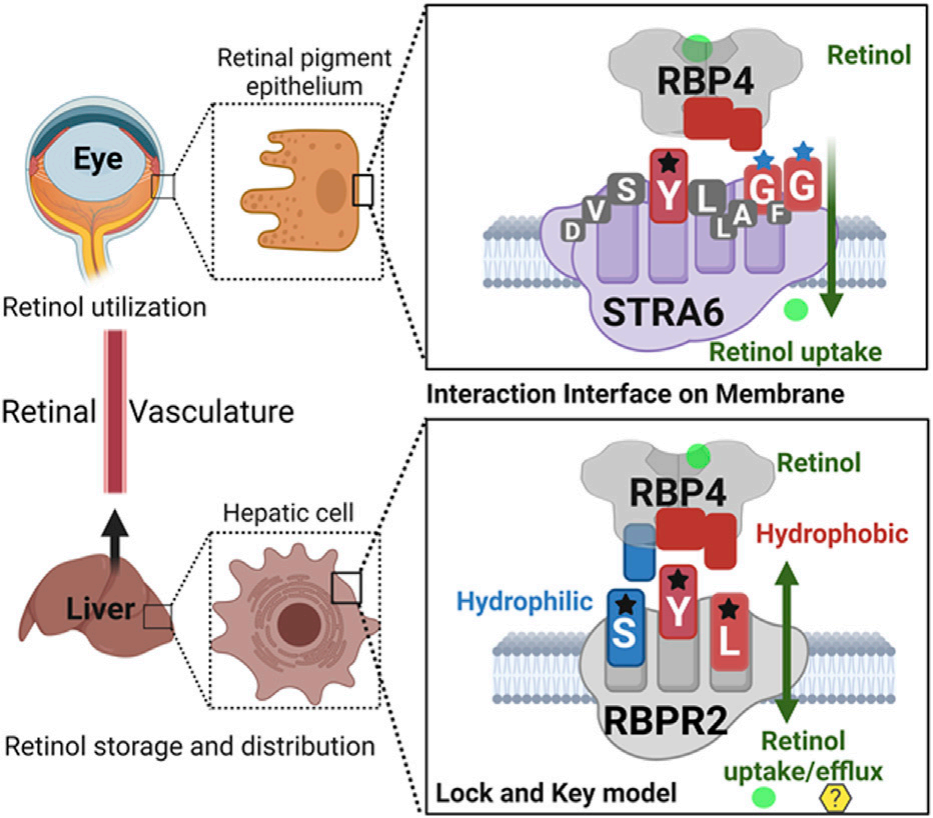
Schematic representation of the proposed RBP4 binding motif on RBPR2 for retinol uptake, storage, and distribution.Shown are the proposed RBP4 amino acid binding residues on STRA6 (YGG) and RBPR2 (SYL) that utilize a lock and key model mechanism to bind and stabilize the extracellular RBP4-retinol complex. The interaction interface between the membrane receptor and its’ ligand is stabilized by both hydrophilic and hydrophobic interactions for retinol internalization. The proposed binding residues for both RBP4-ROL receptors are color-coded based on the property of the amino acid residues.

Given our results and those previously shown by the Sun and von Lintig laboratories, we can speculate that evolutionary vitamin A receptor (s) selection and distribution in a tissue-specific manner provides an advantage in the proper transport, storage, and utilization of all-*trans* retinol in the mammalian system, where retinoids are not a product of *de-novo* synthesis and thus require an active transport mechanism/membrane receptor to reach their target organs (Figure 8) (Chelstowska et al., 2016; Borel and Desmarchelier, 2017; Martin et al., 2021). In almost all mammalian systems, all-*trans*-retinol is the most abundant retinoid in the circulation and would serve as the probable form of retinoid delivered to body systems and would additionally serve as the substrate for the previously discussed membrane receptor. Due to its lipophilic nature, all-*trans*-retinol requires a carrier protein to reach target organs (Quadro et al., 1999; Chelstowska et al., 2016; Borel and Desmarchelier, 2017; Martin et al., 2021). With the discovery of the liver-secreted protein retinol binding protein 4 (RBP4) in 1968 as the specific carrier for retinol and subsequent investigation in *Rbp4* and *Stra6* deficient mice. The mechanism of transport and uptake of retinol to specific target organs can now be elucidated to a further degree (Quadro et al., 1999; Kawaguchi et al., 2007; Amengual et al., 2014; Shen et al., 2016; Borel and Desmarchelier, 2017).

In this study, we hypothesized that the second vitamin A/retinol binding protein 4 (RBP4) receptor, RBPR2, is necessary for the systemic facilitation of dietary retinoid uptake, storage, and transport to targeted organs, specifically the liver and eye (Alapatt et al., 2013). We based our hypothesis on published literature stating that RBPR2 shares amino acid and structural homology with the well-characterized vitamin A/RBP4 receptor, STRA6 (Alapatt et al., 2013; Chen et al., 2016). In the circulation, vitamin A/all-*trans* retinol is bound to RBP4 (RBP4-ROL/holo-RBP4), and on the binding of RBP4 to STRA6, retinol is transported into the cell without internalization of RBP4 (Blomhoff et al., 1990; Kawaguchi et al., 2008a; Isken et al., 2008; Kawaguchi et al., 2008b; Kawaguchi and Sun, 2010; Sun and Kawaguchi, 2011; Sun, 2012; Zhong et al., 2012; Alapatt et al., 2013; Breen et al., 2015; Chelstowska et al., 2016; Shen et al., 2016; Shi et al., 2017; Borel and Desmarchelier, 2017; Lobo et al., 2018; Solanki et al., 2020; Martin et al., 2021; Radhakrishnan et al., 2022a). To understand the mechanism(s) of RBPR2 binding to RBP4, we first employed an *in silico* protein-ligand interaction approach, multiple *in vitro* biochemical assays, and utilized an *Rbpr2* ^*−/−*^ deficient mouse model to predict RBPR2-RBP4 binding characteristics, to assess its empirical binding characteristics, and to observe the physiological consequences of RBPR2 deletion, respectively.

In the past, the Sun lab expanded upon the mechanism (s) of how the membrane expressed vitamin A receptor STRA6 facilitates retinol transport from its carrier protein, RBP4, into cells, where they used an elegant large-scale mutagenesis approach and identified three essential residues on STRA6 that might be essential for RBP4 binding and subsequent ROL transport into target tissues (Kawaguchi et al., 2008a; Kawaguchi et al., 2008b; Kawaguchi and Sun, 2010; Sun and Kawaguchi, 2011; Chen et al., 2016). These residues (Tyr336, Gly340, and Gly342) on STRA6 are highly conserved among mammals and, interestingly, are in close proximity to the proposed “SYL” RBP4 binding domain on the less defined vitamin A receptor, RBPR2 (Figures 1A, 8) (Alapatt et al., 2013). The SYL residues (S294, Y295 and L296) on mouse RBPR2 were previously shown by the Graham group to be of importance through both *in vitro* and CRISPR mutant zebrafish model (s), and are critical for RBP4-ROL binding and retinol uptake and transport, in supporting visual function (Seeliger et al., 1999; Kawaguchi et al., 2008a; Kawaguchi et al., 2008b; Kawaguchi and Sun, 2010; Sun and Kawaguchi, 2011; Alapatt et al., 2013; Shi et al., 2017; Lobo et al., 2018; Martin et al., 2021).

Based on the work of the Sun Lab, we propose that a similar SYL binding motif might also be found in RBPR2, given its similar capability in binding RBP4-ROL (Figure 8) (Radhakrishnan et al., 2022b). Through homology and docking studies, an SYL amino acid consensus was found on the proposed RBP4 binding domain of mouse RBPR2. The importance of the RBPR2 SYL domain was then examined through the mutagenesis of individual residues in the proposed “SYL” binding domain of RBPR2 and by overexpression in NIH3T3 cells, where we observed that all three RBPR2-mutants, like the WT-RBPR2, localized predominantly within the plasma membrane. Subsequent subcellular fractions indicated that individual RBPR2 mutants, like WT-RBPR2, trafficked properly to the plasma membrane (Figure 4). However, in RBP4-vitamin A uptake experiments, all three RBPR2-SYL mutants failed to properly uptake exogenous RBP4-ROL, indicating that these three residues likely contribute to a specific RBP4 binding domain on RBPR2 for ROL transport. However, we were unable to test the combined effects of mutant RBPR2-SYL in a single mutant peptide, as the peptide synthesis and HPLC analysis was not optimal. The calculated binding affinity (*K*_*d*_) of RBP4 for RBPR2 peptides (encompassing the SYL domain) was 25.43 μM for mouse RBPR2 and 33.25 μM for zebrafish RBPR2. The calculated binding affinity (*K*_*d*_) of RBP4 for mouse STRA6 peptide encompassing the previously reported Tyr^336^, Gly^340^, and Gly^342^ residues was 26.73 μM and this was comparable to mouse RBPR2. The binding affinity (*K*_*d*_) values for RBPR2 binding for its ligand RBP4 were comparable to previously published values for STRA6 for its physiological ligand RBP4 (*K*_*d*_ = 22.4 μM). Interestingly, we observed a reduced binding affinity (*K*_*d*_) and stronger dissociation rate (*K*_off_) for the RBPR2 mutants to its ligand RBP4, compared to the WT. In our docking analysis, we observed Ser294 and Tyr295 on RBPR2 to interact with Arg167 on RBP4, while Leu296 RBPR2 was not directly involved in the interaction. Leu296 might play an essential role in structure stabilization for the interaction; if we compare its observed *K*_*d*_ values to the other RBPR2 mutants and WT-RBPR2, it becomes evident that structural stabilization is equally important. The *K*_*d*_ values for RBPR2 peptides interaction with RBP4, S294A = 89.33 μM, Y295P = 34.91 μM, and L296S = 21.30 μM, indicating that the S294A and Y295P mutant peptides required a higher concentration of RBP4 for binding saturation, while the L296S mutant retained a comparable *K*_*d*_ value when compared to WT-RBPR2 (Figures 2C–E; Figures 5–7). The SYL motif on RBPR2 is crucial in RBP4 complex formation and stabilization, with significantly reduced dissociation rates in mutant RBPR2 peptides and RBP4 interactions. Any changes in these residues result in lower dissociation rates, indicating that the natural behavior of the interaction is affected. This suggests a tighter and possibly non-specific binding for the mutant RBPR2 to RBP4, which demands a more detailed study (Supplementary Figure S7). Based on our studies, it would be fascinating to study the serum kinetics of RBP4-ROL binding and uptake in *Rbpr2*-KO mice (Radhakrishnan et al., 2022a), to determine the contribution of RBPR2 for serum and ocular vitamin A homeostasis (Martin et al., 2021). Therefore, in the future, it would be important to determine the crystal or cryoEM structure of RBPR2 to gain further insight into the physiological role of this RBP4-vitamin A receptor in systemic retinoid homeostasis and for visual function. Table 1.

**TABLE 1:** Amino acid sequences of individual RBPR2 peptides used in SPR analysis. The putative mouse RBP4 “SYL” binding residues on RBPR2 and STRA6 are shown in bold. HPLC and Mass spectrophotometry analysis confirmed purity and sizes of individual proteins.

Peptide name	Peptide sequence	Molecular weight	HPLC-purity (%)	Mass spec
**msRbpr2 (42)**	HVRDKLDMFEDKLE**SYL**THMNETGTLTPIILQVKELISVTKG	4845.12	92.14	Confirms
**msStra6 (40)**	SVVPTVQKVRAGINTDV**SYL**LAGFGIVLSEDRQEVVELVK	4329.94	90.84	Confirms
**zebRBPR2 (34)**	DKLDSLKDSLEQIALSCNQTE**S**VFT**YL**IPSINEF	3862.57	95.94	Confirms

## Data Availability

The raw data supporting the conclusions of this article will be made available by the authors, without undue reservation.

## Abbreviations

ROL, all-*trans* retinol; 11-*cis* RAL, 11-*cis* retinaldehyde; RBPR2, Retinol binding protein 4 receptor 2; LRAT, Lecithin:ROL acyltransferase; RA, all-*trans* retinoic acid; STRA6, stimulated by retinoic acid protein 6; RBP4, Retinol binding protein 4; Stra6l, Stimulated by retinoic acid protein 6 like.
