# Supplementary material for: Mapping of the extracellular RBP4 ligand binding domain on the RBPR2 receptor for Vitamin A transport
Source: Front Cell Dev Biol. 2023 Feb 22;11:1105657. doi: 10.3389/fcell.2023.1105657 (PMC9992173; doi:10.3389/fcell.2023.1105657)
Supplement: Supplementary file 1 [file DataSheet1.PDF]

Title: Mapping of the extracellular RBP4 ligand binding domain on the RBPR2 receptor for vitamin A transport

Authors: Rakesh Radhakrishnan[1], Matthias Leung[1], Ashish K. Solanki[2] and Glenn P. Lobo[1,2,3*]

Affiliations: [1]Department of Ophthalmology, University of Minnesota, Lions Research Building, 2001 6th Street SE, Minneapolis, MN 55455, USA. [2]Department of Medicine, Medical University of South Carolina, Charleston, SC 29425. [3]Department of Ophthalmology, Medical University of South Carolina, Charleston, SC 29425.

*** Corresponding Author**
Glenn P. Lobo, Ph.D.
Department of Ophthalmology and Visual Neurosciences
Lions Research Building, Room LRB 225
University of Minnesota
Minneapolis, MN 55455
Tel: (Office) 612-625-5523
(Cell) 952-378-7359
E-mail: lobo0023@umn.edu

Mouse RBP4 recombinant protein expression

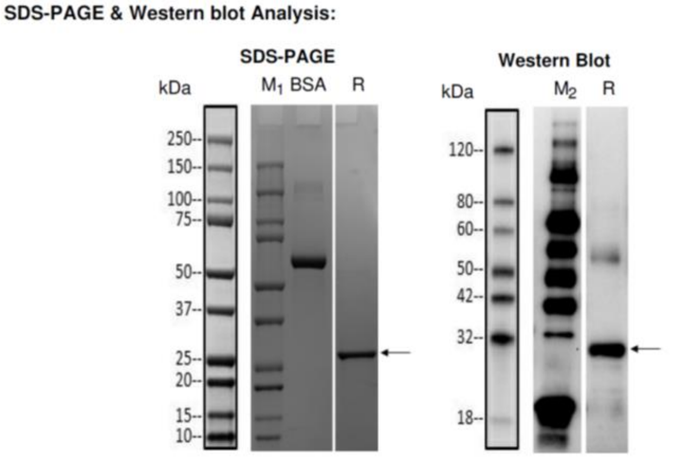

Lane M1: Protein Marker, Bio-rad, Cat. No. 1610374S, refer to annotated key on the left for size
Lane M2: Protein Marker, GenScript, Cat. No. M00521, refer to annotated key on the left for size
BSA: 2.00 µg
R: Reducing condition
Primary antibody: Mouse-anti-His mAb (GenScript, Cat.No. A00186)

Mouse RBP4 sequence

U817SGI150-4_RBP4 mouse **vector: pET30a**

NdeI--ATG--His tag--**RBP4 mouse** --Stop codon--HindIII

Protein Length=260

**MHHHHHHSSGLVPRGSDPLGWRVWLHRDHPERSSEHRRRGTARLTEGPRVRSGGRLRGEMEWVWALVLLAALGGGSAERDCRVSS
FRVKENFDKARFSGLWYAIAKKDPEGLFLQDNIIAEFSVDEKGHMSATAKGRVRLLSNWEVCADMVGTFTDTEDPAKFKMKYWGVASF
LQRGNDDHWIIDTDYDTFALQYSCRLQNLDGTCADSYSFVFSRDPNGLSPETRRLVRQRQEELCLERQYRWIEHNGYCQSRPSRNSL**

Supplementary Figure S1: SDS-PAGE and Western blot analysis of mouse RBP4 protein. Recombinant mouse RBP4 with 6XHis Tag was expressed E.coli expression system and extracted in Tris buffer with a composition of 50 mM Tris-HCl, 1M L-Arginine, 10% Glycerol, pH 8.0. The lysate was purified by a nickel NTA column. The plasmid containing the His tagged mouse RBP4 protein sequence with the used restriction sites for DNA cloning is shown.

Mouse-RBPR2 Peptide Synthesis confirmation

**Date:**	2021-11-26
**Order Number:**	#SP211266
**Product Type:**	Chemically synthesized peptide
**Catalog Number:**	945992
**Peptide Name:**	Mouse Rbpr2
**Sequence (N to C):**	HVRDKLDMFEDKLESYLTHMNETGTLTPIILQVKELISVTKG
**MW:**	4845.12
**Salt Form:**	Trifluoroacetate (TFA Salt)
**Quantity:**	5.6mg
**Suggested Solvent:**	1.0mg peptide soluble in 1.0ml H2O

ASSAY	SPECIFICATION	ACTUAL
MW by MS	4844.50	Conforms
Purity by HPLC	>90%	92.14%
Peptide Content	N/A	N/A

HPLC REPORT

Product Name:Mouse Rbpr2 HG-42
Instrument No: 0200194
Lot No :P211104-CL945992
Column :4.6*250mm C18
Solvent A :0.1%Trifluoroacetic in 100% Acetonitrile
Solvent B :0.1%Trifluoroacetic in 100% Water

Gradient :	A	B
0.01min	30%	70%
25min	55%	45%
25.01min	100%	0%
30.0min	STOP	

Flow rate :1.0ml/min
Wavelength :220nm
Volume: 10µl

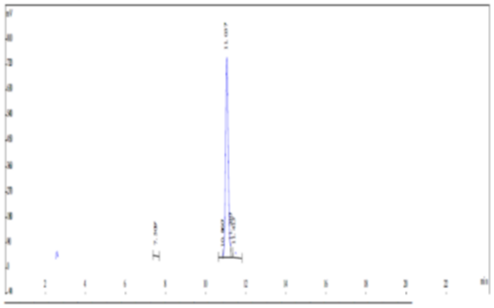

Supplementary Figure S2: Mouse RBPR2 "SYL" peptide synthesis and HPLC purification. The mouse RBPR2 peptide (containing the predicted RBP4 "SYL" binding residues) was chemically synthesized by Biomatik Corporation, Kitchener, ON, Canada. HPLC analysis showing the purity of the peptide.

Zebrafish-RBPR2 Peptide Synthesis confirmation

**Date:**	2021-11-26
**Order Number:**	#SP211266
**Product Type:**	Chemically synthesized peptide
**Catalog Number:**	945998
**Peptide Name:**	Zeb RBPR2 (34)
**Sequence (N to C):**	DKLDSLKDSLEQIALSCNQTESVFTYLIPSINEF
**MW:**	3862.57
**Salt Form:**	Trifluoroacetate (TFA Salt)
**Quantity:**	5.3mg
**Suggested Solvent:**	1.0mg peptide soluble in 1.0ml H2O

ASSAY	SPECIFICATION	ACTUAL
MW by MS	3861.90	Conforms
Purity by HPLC	>90%	95.94%
Peptide Content	N/A	N/A

HPLC REPORT

Sample Description:
Structure:Zeb RBPR2(34)　DF-34
Number: 0200046
Lot No:P211104-CL945998
Column: 4.6mm*250mm, Inertsil ODS-SP
Solvent A: 0.1%Trifluoroacetic in 100% Acetonitrile
Solvent B: 0.1%Trifluoroacetic in 100% Water

Gradient:	A	B
0.01min	37%	63%
25.00min	62%	38%
25.01min	100%	0%
30.00min	Stop	

Flow rate:1.0ml/min
Wavelength:220n
Volume:10μl

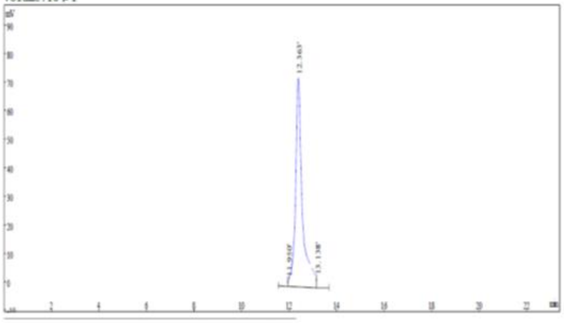

Supplementary Figure S3: Zebrafish RBPR2 "SYL" peptide synthesis and HPLC purification. The zebrafish RBPR2 peptide (containing the predicted RBP4 "SYL" binding residues) was chemically synthesized by Biomatik Corporation, Kitchener, ON, Canada. HPLC analysis showing the purity of the peptide.

Mouse-STRA6 Peptide Synthesis confirmation

**Date:**	2022-02-25
**Order Number:**	#SP220181
**Product Type:**	Chemically synthesized peptide
**Catalog Number:**	968794
**Peptide Name:**	Mouse Stra6 (40)
**Sequence (N to C):**	SVVPTVQKVRAGINTDVSYLLAGFGIVLSEDRQEVVELVK
**MW:**	4329.94
**Salt Form:**	Trifluoroacetate (TFA Salt)
**Quantity:**	5.0mg
**Suggested Solvent:**	1.0mg peptide soluble in 1.0ml DMSO

ASSAY	SPECIFICATION	ACTUAL
MW by MS	4329.25	Conforms
Purity by HPLC	>90%	90.84%
Peptide Content	N/A	N/A
TFA Content	N/A	N/A
Moisture Content	N/A	N/A

HPLC REPORT

Structure :Mouse Stra6 （40）SK-40
Lot NO :P220130-MX968794
Number :0200049
Column :250*4.6mm,Kromasil-C18-5um
Solvent A:0.1%TFA in 100%water
Solvent B:0.1%TFA in 100%acetonitrile

Gradient :	A	B
0.1min	62%	38%
25.0min	37%	63%
25.1min	0%	100%
30.0min	stop	

Flow rate:1.0ml/min
Wavelength(nm):220
Volume :10ul

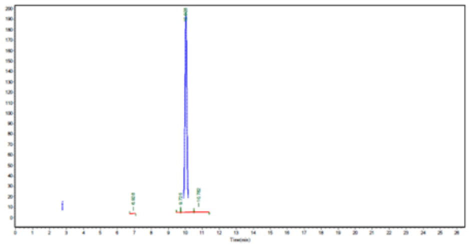

Supplementary Figure S4: Mouse STRA6 “SYL” peptide synthesis and HPLC purification. The mouse STRA6 peptide (containing the predicted RBP4 “SYL” binding residues) was chemically synthesized by Biomatik Corporation, Kitchener, ON, Canada. HPLC analysis showing the purity of the peptide.

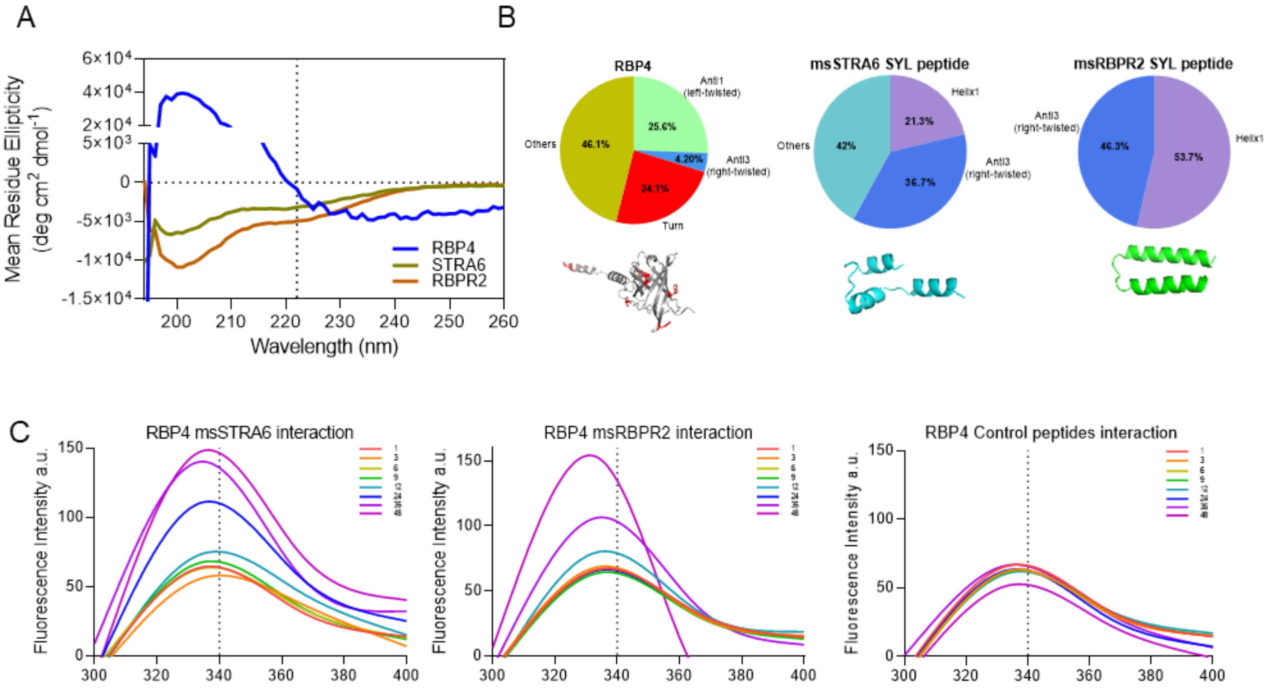

Supplementary Figure S5: The structural composition of RBP4 and STRA6 RBPR2 peptides in Circular dichroism (CD): (A) The plot of RBP4 and RBPR2, STRA6 SYL motif peptides showing the mean residue ellipticity. (B) The pie graph showing the shares of secondary structures present in the molecule. The percent change in molecules structure were calculated using BeStSel Secondary Structure Analysis to Protein Fold Prediction by Circular Dichroism Spectroscopy (https://bestsel.elte.hu) [45,46]. (C) The intrinsic tryptophan fluorescence assay graph showing the interaction of STRA6, RBPR2 and control peptide interaction and change in the fluorescence indicates the ~9

tryptophan residues of RBP4 actively exposed upon interaction.

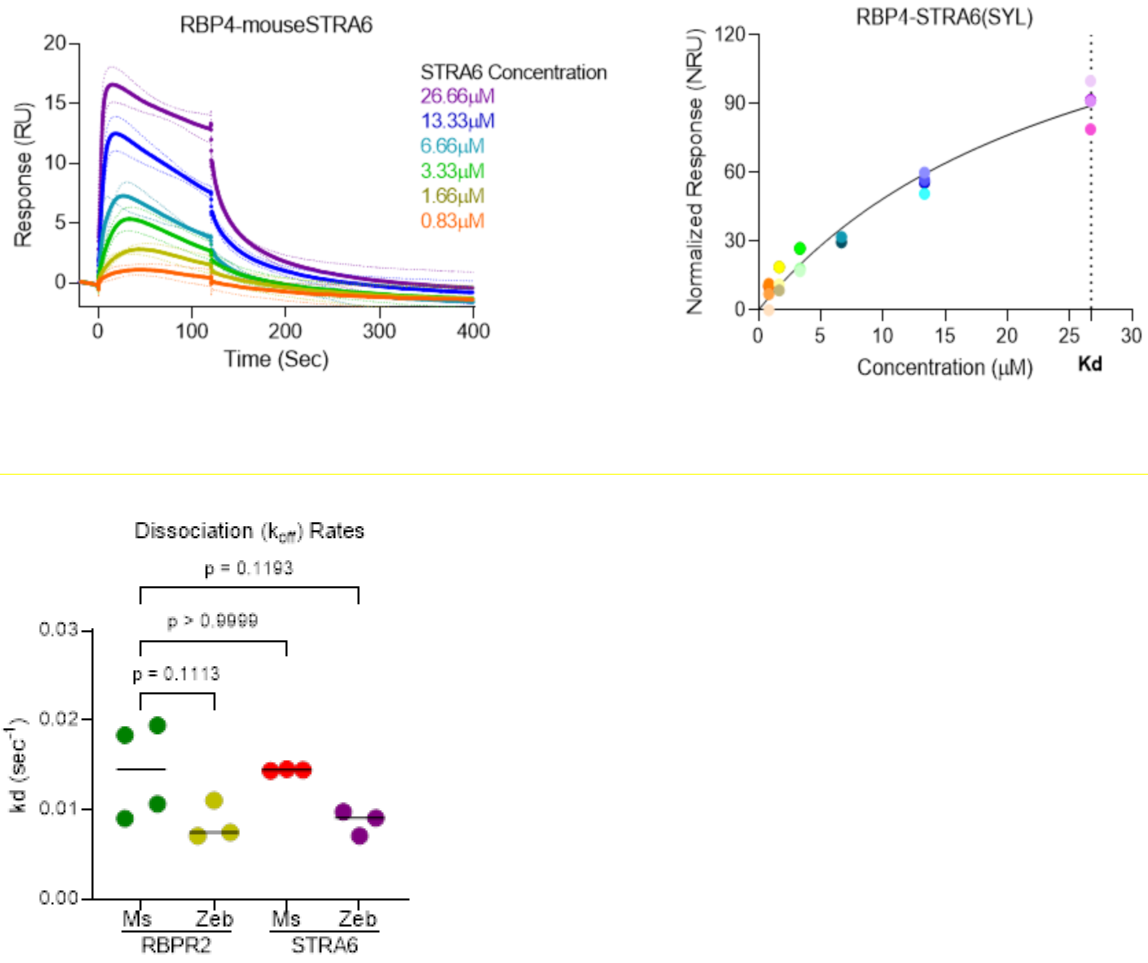

Supplementary Figure S6: Surface Plasmon Resonance binding studies between STRA6 and its ligand RBP4. Binding studies using SPR between mouse STRA6 and immobilized mouse RBP4 protein is shown, together with the respective steady state affinity analysis and kinetic values (K_D) in the right panel. The interaction levels are measured in Response Units (RU) and real time plot sensorgram display the dynamics of the SPR analysis. The dissociation rates showing similar K_{off} values between RBPR2 and STRA6.

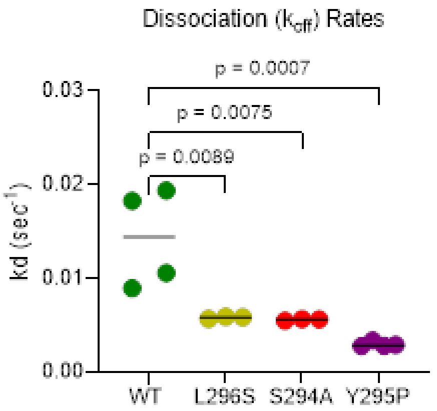

Supplementary Figure S7: RBPR2 mutant peptide interaction rate with its ligand RBP4. The dissociation constant K_{off} value, which implies the strength of the interaction, was significantly stronger in msRBPR2 mutants compared to WT RBPR2.

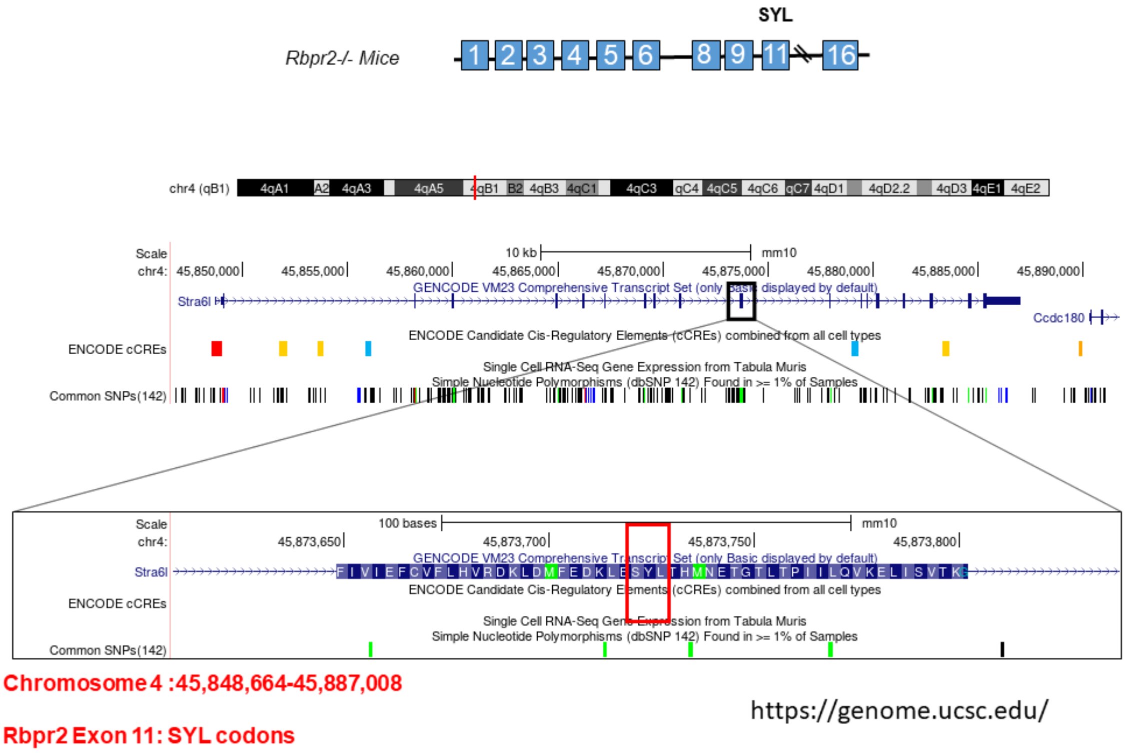

Supplementary Figure S8: Detailed genomic region of mouse chromosome 4 and location of the *Rbpr2* gene. Exon 7 of the mouse Rbpr2 gene was genetically targeted to create an *Rbpr2*$^{-/-}$ deficient mouse [24]. The proposed "SYL" RBP4 binding region/domain is upstream in exon 11 (red box).
